# Community led health promotion to counter stigma and increase trust amongst priority populations: lessons from the 2022–2023 UK mpox outbreak

**DOI:** 10.1186/s12889-024-19176-4

**Published:** 2024-06-19

**Authors:** Colette Pang Biesty, Charlotte Hemingway, James Woolgar, Katrina Taylor, Mark David Lawton, Muhammad Wali Waheed, Dawn Holford, Miriam Taegtmeyer

**Affiliations:** 1 Department of International Public Health, Liverpool University Hospitals NHS Foundation Trust/Liverpool School of Tropical Medicine, Liverpool, UK; 2 Department of International Public Health, Liverpool School of Tropical Medicine, Liverpool, UK; 3 Public Health Department, Liverpool City Council, Liverpool, UK; 4 Sahir House, Liverpool, UK; 5 Liverpool University Hospitals NHS Foundation Trust, Liverpool, UK; 6 School of Psychological Science, University of Bristol, Bristol, UK; 7 Department of Clinical Sciences, Liverpool School of Tropical Medicine, Liverpool, UK

**Keywords:** mpox, public health messaging, participatory health research, pandemic preparedness

## Abstract

**Background:**

Stigma, lack of trust in authorities, and poor knowledge can prevent health-seeking behaviour, worsen physical and mental health, and undermine efforts to control transmission during disease outbreaks. These factors are particularly salient with diseases such as mpox, for which 96% of cases in the 2022–2023 UK outbreak were identified among gay, bisexual, queer and men who have sex with men (MSM). This study explored stigma and health-seeking behaviour in Liverpool through the lens of the recent mpox outbreak.

**Methods:**

Primary sources of data were interviews with national and regional key informants involved in the mpox response, and participatory workshops with priority populations. Workshop recruitment targeted Grindr users (geosocial dating/hookup app) and at risk MSM; immigrant, black and ethnic minority MSM; and male sex workers in Liverpool. Data were analysed using a deductive framework approach, building on the Health Stigma and Discrimination Framework.

**Results:**

Key informant interviews (*n* = 11) and five workshops (*n* = 15) were conducted. There were prevalent reports of anticipated and experienced stigma due to mpox public health messaging alongside high demand and uptake of the mpox vaccine and regular attendance at sexual health clinics. Respondents believed the limited impact of stigma on health-seeking behaviour was due to actions by the LGBTQ + community, the third sector, and local sexual health clinics. Key informants from the LGBTQ + community and primary healthcare felt their collective action to tackle mpox was undermined by central public health authorities citing under-resourcing; a reliance on goodwill; poor communication; and tokenistic engagement. Mpox communication was further challenged by a lack of evidence on disease transmission and risk. This challenge was exacerbated by the impact of the COVID-19 pandemic on the scientific community, public perceptions of infectious disease, and trust in public health authorities.

**Conclusions:**

The LGBTQ + community and local sexual health clinics took crucial actions to counter stigma and support health seeking behaviour during the 2022–2023 UK mpox outbreak. Lessons from rights based and inclusive community-led approaches during outbreaks should be heeded in the UK, working towards more meaningful and timely collaboration between affected communities, primary healthcare, and regional and national public health authorities.

**Supplementary Information:**

The online version contains supplementary material available at 10.1186/s12889-024-19176-4.

## Background

Mpox, originally labelled monkeypox, is a zoonotic viral infection with outbreaks mainly restricted to Central and West Africa. The disease expanded globally in 2022 driven by a new strain, known as Clade II B.1, and increasing human-to-human transmission [1]. As of September 2023, there have been over 80,000 confirmed cases in over 100 countries, with the majority of cases in Europe and North America [2]. Mpox cases were first confirmed in England by the UK Health Security Agency (UKHSA) in May 2022, where the outbreak has disproportionately affected gay, bisexual, queer and other men who have sex with men (MSM). As of December 2023, there have been over 3,800 confirmed cases in the UK [3]. Sexual health services played a key role in the UK’s outbreak response, including case detection and the distribution of preventative vaccines (Modified Vaccinia Ankara (MVA) smallpox vaccine Imvanex) to at-risk groups with multiple sexual partners or participating in group sex [4]. Healthcare providers were instructed to conduct a risk assessment and contact tracing for symptomatic mpox cases, and to advise patients to self-isolate until no longer infectious.

In the early days of the UK mpox outbreak, public health bodies raised concerns over misinformation and potentially stigmatising media reports and online discourse directed at gay, bisexual and other MSM (UKMSM) [5, 6]. Stigma and poor knowledge are a barrier to health-seeking behaviour, which can undermine prevention and response measures during disease outbreaks [7]. Stigma around HIV infections have been found to be associated with less engagement with testing and treatment for the disease [8]. Stigma can be associated with health conditions, lifestyles or directed at groups of people [9]. Internalised stigma (endorsing negative beliefs and feelings about people living with a stigmatised identity and applying those beliefs and feelings to the self) [10], and anticipated stigma (expecting stigma to happen) [11], can be amplified by public health messages that separate people into sexual and behavioural categories, and clinical procedures that reproduce experiences of stigma such as isolation [12]. Given previous evidence, the logical conclusion was that stigma would challenge the mpox outbreak response leading to delayed health-seeking behaviour and diagnosis; unavailable contact tracing details; vaccine hesitancy, and difficulties complying with self-isolation [13]. While the mpox outbreak bore striking similarities with the 1980s HIV epidemic, stark differences such as existing knowledge of the pathogen that causes mpox, lower disease risk, and available diagnostics, prevention, and treatment meant caution should have been taken before assuming the outbreak would face similar challenges [14, 15].

Despite anticipated stigma-related challenges, the UK implemented a successful outbreak response, with national reports showing a dramatic fall in mpox case numbers after a peak in July 2022 [3]. Increased awareness of mpox leading to behaviour modification and high uptake of the Imvamex vaccine among at-risk groups have been identified as leading factors in the decreased transmission potential of mpox [16, 17]. Such evidence indicates the presence of effective public health messages and campaigns able to counter stigma, reduce risky behaviours and encourage vaccine uptake. Yet, commentary on the UK response to mpox suggest success was achieved through LGBTQ + community-led effort in spite of government failings [18, 19].

While community engagement has long been recognised as crucial to address knowledge and stigma-related challenges in infectious disease outbreak responses, there have been calls in recent years to shift away from top-down and potentially exploitive community engagement approaches to more rights based and inclusive community-led approaches [20]. Community engagement on a top-down basis can result in reactive and individualistic responses, shaped by individuals often disconnected from the structures that facilitate and legitimise the stigma in the first place [21]. This understanding has been pivotal in shaping responses to knowledge and stigma-related challenges in the context of COVID-19 in lower- and middle-income contexts, or so called global south [22].

This study sought to compare perspectives from national, regional and community-based stakeholders on the strengths and weaknesses of the UK mpox communication strategy and its influence on experiences of stigma and health-seeking behaviour in Liverpool, UK. Research activities were informed by the Health Stigma and Discrimination Framework by Stangl et al. [12]. The framework articulates the stigmatisation process as it unfolds across the socio-ecological spectrum in the context of health. The domains of the framework connect drivers of stigma with health and social impacts and underpins this process with individuals, organisations and institutions. Findings were used to develop a set of good practice recommendations to inform communication strategies for emerging infectious diseases in the UK.

## Methods

This study used a participatory health research approach, which aims to maximise the participation of those affected by the topic of the research in the research process [23]. Primary sources of data were interviews with key informants selected for their involvement in the mpox response nationally, regionally and in Liverpool, and participatory workshops with priority populations (gay, bisexual, queer and other MSM) in Liverpool.

### Study context

The study compared views of national stakeholders with key informants and priority populations from Liverpool and surrounding areas in the Northwest of England. The region has been credited with hosting the biggest concentration of infectious disease research and development centres in Europe, and is home to one of five High Consequence Infectious Disease (HCID) Units in England, providing access to a broad range of experts [24]. Liverpool City region is said to have ‘*a real sense of place and identity that historically has enabled the population to work together in challenging times*,’ which is reflected in the local authority public health strategy that emphasises collaboration with communities and residents to address health inequalities [25].

At the peak of the outbreak (6 May 2022 to 16 September 2022), the Northwest of England had 216 confirmed or highly probably mpox cases, representing 6.4% of the UK total [26]. It was the third highest region in the national distribution of mpox cases, following from London (69%) and the Southeast (9.1%) [27]. Liverpool City Region faces several context-specific challenges to outbreak response, such as high rates of poverty [28], a large proportion of mobile populations, with the four universities in Liverpool attracting 55,000 students (over 10% of the Liverpool population) [29], and one of the highest concentrations of people seeking asylum per 100,000 population in the UK [30].

The UK mpox response was coordinated by the UK Health Security Agency (UKHSA), who worked with regional and local stakeholders and health providers. Local Liverpool-based organisations involved in the 2022–2023 mpox response included, but were not limited to: Liverpool City Council; Liverpool’s Axess Sexual Health services; Sahir House, a Liverpool based LGBTQ + sexual health charity; Liverpool’s PaSH (Passionate about Sexual Health Partnership); and the Tropical and Infectious Disease Unit in the Liverpool University Hospitals National Health Service (NHS) Foundation Trust. Regional organisations included the Northwest UK Health Security Agency communications team; Northwest NHS communications team; and the LGBT Foundation based in Manchester as a key part of the PaSH network.

### Recruitment

Key informants were identified through consultations with Liverpool City Council and with staff at the Royal Liverpool University Hospital, where mpox cases requiring admission were isolated. Recruitment targeted informants with good insight into the mpox outbreak response and communication strategies. These included clinicians, sexual health service providers, and representatives from third sector organisations (in the UK, this term describes a range of organisations including but not limited to charities, community organisations, and volunteer organisations), and regional and national public health agencies. Purposive recruitment was supplemented with snowball sampling, enabling participants to recommend additional informants. Informants with no relevant expertise, under 18 years old, or not willing to participate were excluded.

For participatory workshops recruitment targeted people disproportionately affected by mpox: users of Grindr and at risk MSM (during recruitment this group were referred to as ‘sex positive’ a non-judgemental term, adopted through consultation with Sahir House, used to support recruitment of people known to the charity that have sex with multiple partners and/or participates in group sex); immigrant, black and ethnic minority MSM; and male sex workers. Immigrant, black and ethnic minority MSM were recruited due to anticipated variation in communication needs and preferences [31]. For inclusion in the workshops, participants had to be male or male identifying, identify as gay, bisexual or MSM, over 18-years old, and currently residing in Liverpool or surrounding regions.

Participants were approached by Sahir House, utilising their support networks. Initial consultations with Sahir House revealed group workshops may not be appropriate with male sex workers and individuals within the immigrant MSM populations due to the risk of unwanted disclosure of sexual behaviour among immigrant groups, and the limited availability and willingness of male sex workers to partake in a full day workshop. Invited participants were therefore given the choice of a shorter one-to-one workshop (~ 1–3 h).

### Data collection

Interviews and workshops were conducted by CB, supported by KT and MWW, under the supervision of CH and MT. Data collection took place between January and November 2023.

Interviews with key informants followed a semi-structured topic guide (see Additional File 1) and were conducted in English virtually or in-person based on participant preference and availability. All interviews were audio-recorded with consent and transcribed verbatim. Interview topics included perceptions of public health messaging and community engagement for mpox; lessons from the HIV and COVID-19 epidemic; and experience and perceptions of health-seeking behaviour, compliance with self-isolation, and vaccine uptake.

Participatory research methods were deployed during workshops with priority populations to stimulate discussion on their experience and perception of the UK mpox outbreak response and elicit infectious disease communication habits, preferences and needs. Workshops followed a detailed facilitators guide (see Additional File 2); methods included charting, group discussion and co-design of a communication campaign for mpox. Participatory workshops were audio-recorded, scatter graphs and participant notes photographed, and contemporaneous notes made by a designated note taker to aid with documentation and reporting. Workshops were conducted in English with access to technological translation services as needed in the migrant, refugee and asylum seeker group. Recordings were transcribed verbatim.

### Analysis

All interview and workshop transcripts were anonymised, with individual identifiers removed to enhance confidentiality. The coding framework was developed deductively using the interview topic guide and the Health Stigma and Discrimination Framework [12], whilst allowing for inductive codes to be identified through reading and re-reading transcripts. CB, CH and MWW independently coded transcripts and then compared and discussed the coding to aid with rigour and trustworthiness of results. Communalities and differences between key informants and workshop participants, as well as between specific priority groups, were identified and triangulated using a framework approach [32]. CB, CH and MT met regularly throughout the analysis process to discuss emerging themes. Study participants and key stakeholders JW, DH, KT, ML were consulted on the findings of the study and asked to review and refine the narrative results until agreement was reached. Study participants were presented with the results of the study using a PowerPoint slide deck and given opportunity to comment. Findings of the study were also presented and discussed at Liverpool City Councils LGBTQ + Health Needs Assessment Working Group, which is attended by representatives from community organisations and primary healthcare providers.

## Results

A total of 11 key informant interviews were conducted (see Table 1) and five workshops (see Table 2).

**Table 1:** Key Informants Interviews (KIIs)

Affiliated Organisation/Sector	Number of Key Informants
UK Health Security Agency	2
Northwest of England Community Organisers	2
Sexual Health Clinicians	2
Infectious Disease Clinicians	4
Media	1
**Total**	**11**

**Table 2:** Workshop Participants

	**Number of Participants**	**Age**
Workshop 1: Migrant, Black and Ethnic Minority MSM	6	30–61
Workshop 2: At Risk MSM	5	37–40
Workshop 3: At Risk MSM	2	Both 58
One-to-one workshop: Former Sex Worker Perspective	1	30
One-to-one workshop: Trans MSM Perspective	1	25
**Total no. of Workshop Participants**	**15**	

Of the workshop participants six reported to have had the mpox vaccine during the outbreak, four had not been vaccinated, and five did not disclose their vaccination status. One participant disclosed a previous mpox infection. Six HIV positive participants chose to disclose their HIV status. In the migrant, black and ethnic minority workshop one participant originated from Algeria, one from Iran, one from Guatemala, two from Nigeria, and one did not disclose their country of origin. Of the key informants, seven identified as male and four as female.

Findings from these interviews and participatory workshops are presented across three distinct themes, with quotations selected to illustrate each theme. Overall, there were both common perspectives and divergent views on the UK mpox communication strategy. There were prevalent reports of anticipated and experienced stigma due to mpox communication and discourse. However, there was consensus among participants that stigma had not significantly impacted on health-seeking behaviour because of collective action by the LGBTQ + community, third sector, and local sexual health clinics. There were mixed views on the effectiveness and delivery of the UK mpox communication strategy. Key informants commended intention by central agencies to collaborate with community-based organisations to develop and distribute mpox messages, but some sexual health and third sector key informants expressed criticism citing under-resourcing; a reliance on goodwill; poor communication; and tokenistic engagement. Mpox communication was further challenged by a lack of evidence on disease transmission and risk, and this challenge was exacerbated by the impact of the COVID-19 epidemic on the scientific community, public perceptions of infectious disease, and trust in public health authorities.

### Collective action to address misinformation & stigma by priority populations & local organisations

A majority of reported mpox cases in the UK were among sexually active gay, bisexual and other MSM, of white ethnicity, aged between 30–40-years-old (UKMSM). Key informants discussed how they felt this particular group of UKMSM had contributed to the rapid decline in mpox cases observed in 2022. They attributed this to the legacy of the HIV epidemic, as respondents described a community experienced in health advocacy, with high levels of sexual health literacy and service uptake, a strong sense of collective responsibility, low vaccine hesitancy, and an open and resourced culture of peer-to-peer support. These qualities were reflected in the experiences of workshop participants, with many reporting to have heard about mpox through friends or sexual health clinics, along with reports of peer-to-peer encouragement to get vaccinated and a sense of duty to protect the progress made in HIV communication.

Respondents provided anecdotes of priority populations travelling to other regions from the one they resided in for the vaccine, advocating for better access to vaccines and sharing information on vaccine availability across multiple platforms. The mpox response in Liverpool was reportedly challenged by a slow and under-resourced national vaccination campaign resulting in supply shortages and restricted access; limited diagnostic capacity and slow turnaround of results were also noted from regional informants but less prominent. Several respondents, including key informants, questioned whether vaccine supply shortages were an act of discrimination against the LGBTQ + community. Demands for the vaccine were angry in tone as this typical quote illustrates:

*“And we need more vaccines. Yes, I want more vaccines. I just think it’s absolutely abhorrent that after COVID, they didn’t see this coming. But then do they care? Because it’s the gay community, one has to ask that question.” – Workshop 2, Respondent 3*

Reflections by a senior health worker suggested such vocal demands for prevention were unique to the mpox outbreak.

*“But yeah, I mean, it was angry, angry, angry messages. ‘I can’t get an appointment for my monkeypox vaccine’. ‘When are you going to have more vaccines in?’, and ‘it’s a disgrace that the vaccine isn’t available’. But we’ve never had that with condoms and HIV prevention or gonorrhoea prevention.” – Senior Sexual Health Consultant_1*

Other respondents drew comparisons with demands for the mpox vaccine and demands for HIV pre-exposure prophylaxis (PrEP).

*“...a lot of people in the GBMSM community wanted the vaccine, wanted to get vaccinated, wanted to get protected. And that’s the same with HIV, you know, that the MSM community were asking for PrEP well before it was available…we’ve seen it happens, we know this works, we want to protect ourselves, give us the tools to protect ourselves. But then, that’s perhaps the well-educated white male segment of the population.” – Infectious Disease Clinician_1*

A majority of the workshop participants had either received or were accepting of the mpox vaccine. Only two participants expressed a degree of hesitancy due to low levels of trust in the pharmaceutical industry and low perceived risk of mpox. Stated regional barriers to vaccine uptake included the online booking system and restrictive eligibility criteria, which again the community reportedly mobilised to help others circumvent. High vaccine demand and uptake appeared coupled with notions of collective responsibility and peer-to-peer encouragement to get vaccinated. Key informants who attended the 2022 Manchester Pride event provided anecdotes of people requesting to be vaccinated on their forearm as a clear signal to others that they were protected against mpox.

Respondents reportedly witnessed a resurgence in fear and discrimination directed towards the LGBTQ + community because of the mpox outbreak and associated sensationalised media reports and stigmatising online discourse. A trans MSM shared his experience of posting about mpox on social media channels:

*“Because I remember I was getting a lot of abuse online because I posted quite a lot about it [mpox]. Like, saying look if you’re affected by this, my heart goes out. And I got a lot of trolls... [saying] you deserve it, thinking that I had the disease myself.” – Trans MSM*

Key informants perceived a degree of resilience among affected communities, enabling them to act on a sense of duty to address and counter stigmatising and misleading information.

### Engagement of sexual health services and third sector

LGBTQ + charities, community groups and sexual health clinics were reportedly tasked with disseminating mpox information by central agencies and perceived to be effective in countering issues of trust, stigma, and restricted access to health services. The walk-in open access and outreach format of sexual health clinics was credited as a key factor in the high uptake of the mpox vaccine. Workshop participants often reported becoming aware of mpox through attending sexual health services and positioned local charities and members of their community as their most trusted source of information.

UKHSA informants spoke of the importance of disseminating consistent messages through locally recognised organisations and individuals, citing an observed decrease in public trust toward central government agencies associated with the COVID-19 response. Many workshop participants corroborated this lack of trust in government since the UK’s COVID-19 response. Migrant workshop participants tended to express neutral positions about the UK government, with some voicing mistrust in governments abroad, especially in the dissemination of public health information as a form of propaganda. UKHSA strategies were guided by behaviour change principles from literature on crisis and risk communication, noting the importance of listening to community organisations and priority populations to identify communication needs and assess the ongoing strategy:

*"As an organisation, obviously we can talk to the data...but actually in terms of the messaging and how it would land…absolutely we [UKHSA] worked with the local partners and national partners to get that messaging right."- UKHSA Informant*

While many key informants commended the UKHSA’s intentions and effort to engage LGBTQ + charities and community organisations, some sexual health and third sector key informants expressed criticism, citing under-resourcing, a reliance on goodwill, poor communication, and tokenistic engagement. Key informants described how short notice instruction from central agencies to distribute and promote vaccines, whilst simultaneously facing supply shortages, undermined the trust their organisation had established in the community, as illustrated by this typical quote:

*“Information was almost released to the public before local systems had had a chance to catch up with it…there were national outlets saying vaccines are available, speak to your local clinic…I feel like it does more harm than good if I'm doing all my outreach and speaking with loads of queer people in Manchester and saying oh vaccines are available, and then they call the clinic and clinic say actually we're not 100% sure what we're doing with it yet...It just breaks down the trust a little bit between people and services.” - Community Organiser*

Some informants felt central agencies didn’t heed their warnings of supply shortages and access issues, and erroneously assumed there would be low demand for the vaccine.

Communication assets developed by central agencies, while commended for using simple and concise language, were at times described as stigmatising, generic, and neither engaging nor persuasive. Participants did not recognise, nor had they previously witnessed UKHSA- and NHS-branded mpox messages shown to them during workshops. A key informant felt government bodies could have made better use of social media platforms to disseminate and target mpox messaging, comparing mpox messaging on their social media with COVID-19:

*“I saw [mpox messaging] shared from other people, but I saw very little. And with COVID it was everywhere. It was on my timeline from government sources, in terms of government sources had used digital advertising money to put it on my timeline. Which they could have very easily done with monkeypox and targeted towards gay queer bi people.” – Media Specialist*

This was countered by other key informants who warned against over-communication resulting in distorted public perceptions on risk and information fatigue.

*“I sometimes wonder, and this was more to do with COVID, but whether we sometimes over communicate…and you end up thinking that COVID is the only thing in our lives and it becomes all-encompassing…I think the level of communication, the amount of communication, they [public health authorities] could think about that a bit more. I did wonder whether we overwhelmed people with information at some points during COVID, don't think that happened with monkeypox so much.” – Infectious Disease Clinician_2*

Local third sector and sexual health informants felt that instruction by central agencies to target sex-on-premise venues were misguided, citing a shift in group sex activities to private venues facilitated through social media, and established safe sex policies in commercial venues. Considering reported shortcomings of mpox communication by central agencies, some community organisation informants felt opportunities to co-develop messages and communication strategies with affected communities were missed. They took it upon themselves to design their own communication strategy and assets in a way they felt would resonate better with the local community, e.g., by using local colloquialisms. Workshop participants reported local colloquialisms, together with humour in messages, helped facilitate engagement especially in a post-pandemic context where participants were fatigued by public health and outbreak communications.

### Limitations & challenges in evidence-based public health messaging

A prominent challenge in generating public health messages for mpox was the need to strike a balance between providing clear, non-stigmatising, actionable information, while being open about uncertainty and avoiding under- or overstating risk. This challenge was further exacerbated by limited evidence on the transmission dynamics of Clade II B.1 and the virus’s history as a neglected tropical disease in Central and West Africa. Key informants witnessed speculations about sexual transmission of mpox in parts of Asia and Africa prior to the 2022 outbreak and questioned whether we would have been better prepared for the UK outbreak had mpox been *‘as well funded as other high-profile diseases.’*

Key informants believed the post COVID-19 era had heightened the importance of providing accurate evidence-based information while avoiding speculation. For example, an infectious disease clinician described how groups of social media users remained primed to leverage scientific communication to suit their own agendas, which led them to question whether communication of disease risk may have been downplayed:

*“With hindsight I wonder did that lead us to downplaying in some ways the risk, because we knew that if we even hinted the smallest idea, that it could be really magnified and taken out of context…[in reference to their publication on mpox] The majority of the tweets were conspiracy tweets, it was hashtag monkeypox is airborne…which is not what we said at all but they took it from where we were speculating, monkeypox can be perhaps transmitted by respiratory droplets in short distances and they were taking that and blowing that out of proportion.” – Infectious Disease Clinician_1*

UKHSA key informants felt one of the key challenges in accurate health communication was that people tend to find definitive information clearer and more trustworthy, whereas messages containing nuance and uncertainty are less accessible and believable. Workshop participants described being cynical towards information on the internet, especially if it appeared to have an agenda, be it political or commercial. They highlighted the importance of honesty and demonstrated preference for health authorities to be upfront about unknowns.

A reported positive outcome of the mpox outbreak was how it motivated research into a previously neglected tropical disease to inform practice. Central agencies were commended for responding quickly to new research findings, for example, refining self-isolation messaging based on emerging evidence on the transmission dynamics of Clade II B.1. Shortened self-isolation periods were welcomed by the workshop participants, who were fearful of further stigmatisation from prolonged isolation from important social groups and already fatigued by COVID-19 lockdown measures.

Key informants discussed how evidence on disease burden and risk influenced the target audience for mpox messages. Respondents were cognisant that regular uptake of sexual health testing services was highest among openly gay, bisexual, white British ethnic, well-educated men or trans men. Questions were raised as to whether targeting activities based on the demographics of reported mpox cases meant other at-risk groups who do not regularly engage in sexual health services, namely sexually active heterosexuals, discrete MSM, and non-white British MSM, were overlooked in communication and outreach activities in the mpox response:

*“…was it a huge spike in actual cases, or a large spike in recognition and reporting of cases? Whereas maybe a heterosexual man who got a spot on his willie didn't go to a clinic didn't think anything about it, and it settled down on its own?” – Senior Sexual Health Consultant_1*

*"People who are coming from other communities or are more closeted during the transmission. You know, there are a lot of people who didn't admit to any known links, which probably means that either through anonymous sex or not being out…that probably facilitated the spread early on, and our messaging…isn't going to get through to that less visible GBMSM community" – Infectious Disease Clinician_1*

Key informants spoke of a difficult balance between using data to direct limited resources to at-risk groups and the potential to generate negative associations between disease and sexual identity. Some key informants felt targeting messages at UKMSM was ultimately the right approach. Among priority populations interviewed there was a consensus that promotion of mpox diagnosis and prevention should be broadly inclusive, and risk should be communicated in terms of behaviour, not sexual identity. Perceptions from both key informants and priority populations were underpinned by awareness and experience of HIV/AIDS communication in the 80s and its social legacy. Some respondents believed if a population or community needs to be labelled as ‘at-risk’ for purposes of awareness, identification and diagnosis, then there needs to be clear explanation to the public as to why. This would help to disentangle identity from behaviours and reduce stigma. One participant explained similarities in the shortcomings of HIV communications, where clear explanations as to why people who are at-risk were lacking:

*"..in my country there's information about HIV as well. And they tend to say like, ‘hey people, men that sleep with other men are high risk’, [but] they never explain why. And a lot of people use that information, outside of the gay community as well, inside as well, to shame the gays or the gay community…[public health organisations] never make an effort to explain why gay people are more at risk than straight people. – Workshop 1, Respondent 6*

There was a common sentiment across respondents, that given the right information, people are generally willing to behave in a way that is protective of their health and the health of their community. This was associated with a preference for anti-paternalistic messages and non-judgemental promotion of sexual health services.

## Discussion

**Fig. 1:**
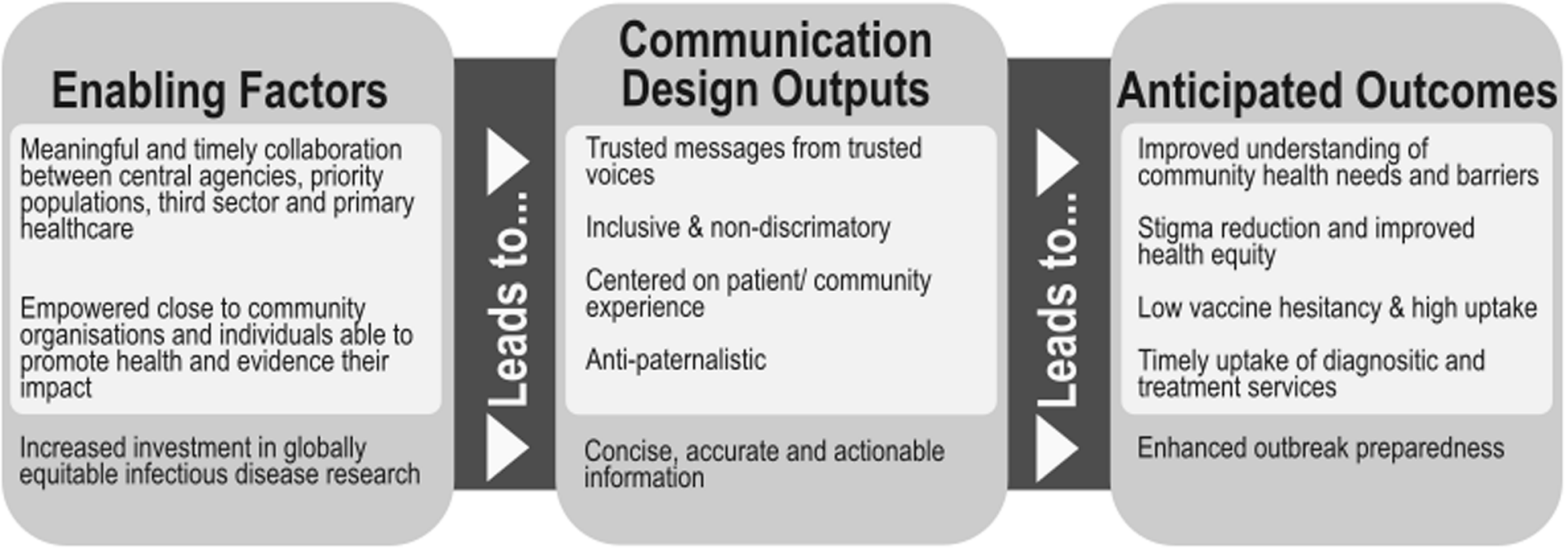
Logic model for community led infectious disease communication

Drawing on the study findings, Fig. 1 presents a logic model for public health communication strategies in disease outbreaks. The logic model summarises perceived enabling factors for effective health communication, communication design outputs, and anticipated outcomes on health-related knowledge and behaviour, as described by the key informants and priority populations recruited for this study. Components of the model are then discussed in reference to the literature.

### Enabling factors

LGBTQ + community organisations and local sexual health services played an essential role in the 2022–2023 UK mpox response. These organisations were able to foster and support a community experienced in health advocacy, with high levels of sexual health literacy and service uptake, a strong sense of collective responsibility, low vaccine hesitancy, and a culture of peer-to-peer support. Organisations were primed and experienced in delivering non-stigmatising health information to GBMSM due to their historic and ongoing role in the HIV epidemic, enabling them to rapidly mobilise and translate emerging mpox knowledge into co-produced advocacy and health promotion campaigns. The organisational legacy of the HIV epidemic in shaping the mpox response is not unique to the UK, with similar experiences evidenced in Australia [33]. Researchers have been calling for timely and meaningful engagement with at-risk priority populations and community organisations since the lessons learnt from the 2013–2016 West African Ebola outbreak [34–36]. Inclusion of community organisations by UKHSA and other central public health agencies was praised, however key informants report further to go to achieve meaningful two-way collaboration between communities and public health authorities. Key informant description of community engagement by central public health agencies was reflective of a top-down reactive and individualistic approach, which has been criticised for failing to fully incorporate the lived experience of affected communities in outbreak response activities [21, 22]. These findings are reflective of typical community engagement approaches in high-income countries, where community engagement used for infectious disease prevention and control during epidemics is largely limited to consultation, demonstrating passive involvement with target communities [37].

A paper reflecting on New York’s community-led response to mpox affirms ‘*power to make consequential decisions should be placed in the hands of those whose lives will be affected by those decisions’* [38]. Liverpool possesses a rich context of collaboration and power sharing in public health. A cross sector LGBTQ + Health Needs Assessment Working group, consisting of academics, LGBTQ + charities, youth groups, sexual & reproductive health services and other community organisations, commissioned by Liverpool City Council, conducts participatory research to better understand the health needs of the LGBTQ + community and improve health equity. Liverpool City Council have invested in community-led models to improve vaccination (COVID-19, childhood measles, mumps and rubella) and cancer screening uptake, by empowering and resourcing interdisciplinary teams consisting of health practitioners, community organisations and priority populations to use data to develop solutions to complex public health challenges such as issues of trust and misinformation. In conclusion, the Liverpool context provides a wealth of opportunity to address gaps in resources and know-how to develop effective outbreak responses with affected communities. However, efforts to sustain and scale-up community-led health promotion and collective action against health inequities is challenged by reactive and siloed funding streams which result in the loss of networks and duplication of resources, an experience reflected by key informants who felt time was wasted through having to re-establish working groups for each emerging public health crisis.

Engaging communities, community organisations, and sexual health services in outbreak responses should not lead to burdening them. Resources are required to match the role community organisations and sexual health services are expected to play. Reports of under-resourcing and poor communication by central agencies placing strain on sexual health services was not unique to the Liverpool context, a 2022 survey of 139 UK sexual health professionals with direct clinical experience of mpox found increased workload pressures were exacerbated by a lack of additional funding for mpox, pre-existing pressures on sexual health services, and unrealistic expectations around capacity, resulting in 67.6% of respondents reporting negative emotional impact due to their mpox work [39]. Across different contexts, the 2022–2023 mpox outbreak put increased strain on sexual health services, while opportunities to strengthen sexual health service capacity to better meet the health needs of the local community were missed [40]. This is potentially reflective of sentiments by key informants, who appreciated being invited to the table but didn’t feel heard when expressing the needs of local sexual health services and the community. A further missed opportunity centred around co-development of mpox communication between central agencies and affected communities, enabling a shift from the traditional top-down information-based model of health promotion to a more grounded narrative approach, utilising patient experiences and storytelling to help communities make sense of the uncertainty and novel situation brought about by infectious disease outbreaks [41]. In a promising step towards addressing missed opportunities, the UKHSA recently announced a £200,000 fund to award innovative community-based organisation campaigns that boost engagement through outreach activities to reduce sexual health inequalities in LGBTQ + communities [42].

Underpinning effective infectious disease outbreak communication are accurate evidence-based messages, without which uncertainty becomes a significant challenge for community-based messengers, undermining their trust and ability to promote positive health behaviours. The nature of health research means uncertainty will almost always be present, in these circumstances workshop participants recommended honesty and transparency. Research on the impact of scientific uncertainty on trust and behaviour change is limited and has shown mixed results depending on how the uncertainty is presented and who it is presented to [43]. In addition to issues of uncertainty, the ability to accurately explain risk in terms of behaviours requires a good understanding of disease transmission dynamics. Key informants and workshop participants perceived this type of messaging to be important to reduce manifestations of stigma due to conflation of risky behaviour with certain identities. Generating a strong evidence base requires research in all countries where the disease is endemic. Cessation of mpox’s WHO designation of a ‘Public Health Emergency of International Concern’ brings with it a risk of complacency and reduced investment in research and development, especially in resource constrained countries where mpox remains a significant ongoing threat [44]. Promisingly, the first clinical trial on the African continent related to a mpox therapeutic antiviral, Tecovirimat, is due to begin in the Democratic Republic of Congo due in part to the 2022 global outbreak [45].

### Communication design outputs

Enabling factors support development and delivery of preferential communication design attributes and encompass the message content and execution. Study respondents voiced preferences on message content to be concise and factual; unambiguous language; transparent and honest about evidence base; risk explained in terms of behaviour not social identity; and utilising patient perspectives. Tone of the message content should be anti-paternalistic, guiding people to assess their own risk, with clear instruction on prevention, diagnosis and treatment. These findings are reflective of twenty-first century advances in risk and crisis communication which emphasise public involvement and ‘dialogues free of prejudices, paternalism, and preconceptions…[to]…impart precise and updated information reflecting uncertainty and considering cultural differences to build trust and facilitate cooperation with the public sphere [46].’ Despite anticipated variation among migrant, black and ethnic minority population, reported communication needs and preferences were consistent across the workshops and no cultural differences were identified beyond a need to provide information in multiple languages and preference for images over text to support accessibility.

Respondent preferences for message execution included accessibility to all people with varied communication needs; delivery through multiple avenues online and offline; with layered tiers of information to avoid over- or under -communication. In the United States, dissemination of information through locally recognised trusted messengers was pertinent to the successful mpox response and stigma reduction [47]. The engagement of community organisations when designing public health messaging has proven to be especially essential when working in a context of mistrust between communities, outbreak responders, and government [48]. Trust in the UK government has been on the decline, exacerbated by political scandals of governmental officials breaching lockdown rules [49]. Lack of public trust in government can undermine messaging from public health authorities [50].

### Anticipated outcomes

The study indicated that empowered affected communities were pertinent to the UK’s successful outbreak response. These communities were able and willing to share mpox information and patient experience, advocate for improved access to health services, counter stigma and misinformation, and encourage vaccine uptake among peers. Hypothesised stigma associated reduced health-seeking behaviour, delayed presentation or vaccine hesitancy [5, 6] was not found among participants and key informants in the Liverpool experience of the mpox outbreak. Moreover, many participants and key informants described how priority populations were eager to get the mpox vaccine, but faced access barriers to vaccination. This corresponds to mpox vaccine willingness in other settings such as in the Netherlands [51] and the United States [52], and contrasts with the significant vaccine hesitancy underpinning the COVID-19 vaccination programme [53]. This correspondence and divergence from literature on vaccine uptake, which may be influenced by differences in health literacy and prevailing attitudes among the at-risk population, speaks to the importance of these factors for the effectiveness of community-led health promotion. Thus, the logic model may not be transferable to other disease outbreaks without adaptation that considers important characteristics of the at-risk population. In some circumstances, infectious disease communication can be impactful in its simplest form: communicate the disease risk, and provide access to prevention and treatment, and individuals will implement these short-term solutions to return the body to homeostasis. However, as this study shows, the ethos of the simplistic approach will often fall short of delivering an equitable response to disease outbreaks because it fails to acknowledge the lived experiences of marginalised and disadvantaged groups and their needs for trusted sources of communication and healthcare provision. Communication from culturally-connected, trusted sources, that responds to individual community needs and barriers to healthcare, can help dismantle health inequities while providing clear instruction on prevention and treatment services [54].

### Limitations

This study focusses on the Liverpool experience of mpox in the 2022–2023 outbreak. Although themes of communication good practice recommendations could be transferrable, it may be difficult to generalise this study to other outbreaks with different contextual challenges e.g., countries with human rights violations. Recruitment limitations meant younger and student MSM demographics were not well represented in the data, but they were potentially a key sub-group. Another potentially important group not represented in the data were closeted and discreet MSM. Reports that migrant, black and ethnic minority MSM faced multiple forms of discrimination due to their intersecting minority identities and thus had a greater subgroup of closeted or discreet MSM [55] informed expectations that there would be variation in communication needs and preferences among migrant, black and ethnic minority MSM. However, the lack of representation of closeted and discreet MSM in the data may explain why reported communication needs and preferences were consistent between the workshops with different MSM groups. Sahir House led on recruitment and provided participant reimbursement raising the possibility of bias in responses to favour charities and community organisations. Participants were not directly asked if they had previously acquired mpox. It was not deemed ethical to encourage participants into revealing sensitive health information during the workshops as their confidentiality could not be guaranteed, however it is acknowledged that this information could have been gathered using other methods. This could be a limitation, since lived experience of an illness can shape and individuals’ perception and experience of stigma [56. We do not know if bisexual participants were represented in the workshops, which is another factor that could shape mpox vaccine willingness [57]. Finally, when assessing the high demand for the mpox vaccine, there may be other factors than those outlined in this paper, such as perceived scarcity, which was shown to increase demand for COVID-19 vaccines in Germany [58].

## Conclusions

The 2022–2023 UK mpox outbreak has shown the necessity of co-developing public health messaging with affected priority populations to produce persuasive, accessible, informative and non-stigmatising public health communications. The UK response to mpox shows some learning from previous public health campaigns around HIV and COVID-19, but key informants expressed room for improvement. Lessons from rights based and inclusive community-led approaches during outbreaks should be heeded in the UK, working towards more meaningful and timely collaboration between affected communities, primary healthcare, and regional and national public health authorities.

### Supplementary Information

Supplementary Material 1.

Supplementary Material 2.

## Data Availability

The datasets generated and/or analysed during the current study are available from the corresponding author on reasonable request. Assessment instruments and topics guides are included as additional files.

## Abbreviations

UKMSM: Reported priority population for mpox in the UK
MSM: Men who have sex with men
UKHSA: UK Health Security Agency
