# Supplementary material for: Community led health promotion to counter stigma and increase trust amongst priority populations: lessons from the 2022–2023 UK mpox outbreak
Source: BMC Public Health. 2024 Jun 19;24:1638. doi: 10.1186/s12889-024-19176-4 (PMC11188168; doi:10.1186/s12889-024-19176-4)
Supplement: Supplementary file 1 — Supplementary Material 1. [file 12889_2024_19176_MOESM1_ESM.docx]

**Appendix 1: KII Topic Guide**

*Section 1: Opening questions*

1. Tell me about your roles during the response to MPX?
2. When and how did you find out about MPX? that MPX was mostly affecting MSM?

*Section 2: Public health messaging This project analyses the role of messaging and how it can be improved*

1. Are there any messages or information you wish had been delivered better or earlier? Why?
2. How did you feel the messaging portrayed MSM?
3. How well were messages received by MSM?
   1. Probe: messages about self-isolation? messages about vaccination? messages about social distancing? Do you think they were heeded?
4. There was a focus on how MPX is not a sexually transmitted infection. How was this received?: Could there have been pros and cons to this approach?
5. What phrase would you like to have heard less of? What phrase would you like to have heard more of?
6. After experiencing Covid and the importance of public health messaging, do you feel there have been lessons learnt from this?
7. Looking at the MPX messaging, have there been any lessons learnt / techniques adapted from public health messaging around HIV?

*Section 3: Presentation of healthcare*

1. Have you experienced communication as a barrier to presentation?
2. Did you experience late presentations as part of your work?
3. How did the public health messaging support presentation to services? How might it have hindered?

*Section 4: Self isolation*

1. [as appropriate] You said earlier your role involved talking to people about self-isolation. In your practice did you feel the existing public health messages made your role easier?
   1. Probe: How did it make your role easier?
   2. Probe: How did it make your role harder?
2. How did information circulating on social media affect your conversations with people?
3. How did you convey messages?
   1. Probe: What was most effective?
   2. Probe: How did you avoid stigmatising the infection?

*Section 5: Vaccination*

1. What sort of communication was there surrounding the MPX vaccine?
   1. Probe: Did this have any effect on whether people consented to the vaccine
2. What are some opinions around the vaccine?
3. What communication techniques can be used to reduce barriers and improve patient outcomes / uptake of vaccination?

*Section 6: Summary*

1. Are there any recommendations you have to make to PHE about communication around MPX or future outbreaks?
   1. Probe: What about local organisations like Sahir house and/or Liverpool Pride?
2. Is there anyone else I should be interviewing/talking to with an interest in stigma and health care?
3. We will be doing interviews with a range of different MSM. Are there any particular areas we need to explore in these?
