# Supplementary material for: Community led health promotion to counter stigma and increase trust amongst priority populations: lessons from the 2022–2023 UK mpox outbreak
Source: BMC Public Health. 2024 Jun 19;24:1638. doi: 10.1186/s12889-024-19176-4 (PMC11188168; doi:10.1186/s12889-024-19176-4)
Supplement: Supplementary file 2 — Supplementary Material 2. [file 12889_2024_19176_MOESM2_ESM.docx]

**Appendix 2: Workshop Plan**

**Venue:** [to be agreed with the participants – LSTM teaching or meeting rooms will be used where acceptable]

**Date & time:** [TBC]

**Participating organisation(s):** [refer to section on workshop recruitment in protocol]

**Lead facilitators:** Colette Biesty, Charlotte Hemingway

**Note taker:** [TBC]

**Scatter graph activity:**

- Large felt board or white board
- Tape (double sided) or Velcro
- X & Y axis labels (laminated)
- Blue tack/ stickers/ marker

Participants will use markers or stickers to temporarily indicate their level of agreement to different statements on the X & Y axis. The facilitator will prompt participants to explain/ discuss their response and explore divergent views. The note taker will photograph each completed scatter graph before moving on to the next question.

**Workshop schedule:**

Aim for 5-minute breaks between activities, time permitting.

| **Activity** | **Time** | **Facilitator notes** | **Target output** |
| --- | --- | --- | --- |
| Project introduction | 09:00 – 09:30 | Short presentation introducing the project and workshop objectives.  Confirm all participants have provided written informed consent.  Agreement on workshop code of conduct. | Shared understanding of workshop objectives |
| Participant introductions | 09:30 – 10:00 | Instruct participants to introduce themselves stating their name [or nickname] and age.  As participants give their introductions ask them:  Tell us about a news article/ advert/ message you’ve seen about Mpox?   - Probe: why did you choose to talk about that article/ advert/ message? - Probe: how would you rate it? Good/bad, informative | - Participant demographics - Mpox communication habits, perceptions insight |
| Icebreaker/ refreshments | 10:00 – 10:30 | Begin by having participants pick a partner. Have the pair sit back-to-back. One participant has a blank pad and pencil, while the other is given a picture with an obscure shape on it. The participant who’s holding the picture instructs the one with the pad on what to draw.  The goal is to see how accurate the resulting drawing turns out. This icebreaker helps participants learn to rely on each other and communicate in a detailed manner. | Trust building |
| Scatter graph activity: comms reel | 10:30 – 12:00 | Discuss each slide from the ‘Workshop Comms Reel’ slide deck using the scatter graphs. Continue discussion until there are no new views being expressed.  Each slide contains an organisation/ media outlet/ social media platform that has disseminated information about Mpox and a link to a webpage containing the latest news articles/ information on Mpox.  For news organisations, select 2 or more news articles to discuss.  For social media platforms, select 2 or more posts/ message boards to discuss.  Complete the following scatter graph discussions for each slide:  **Potential for stigma**  X axis – how accurate is the information? Very – Not at all  Y axis – how stigmatising is the language/ images? Very – Not at all   - Probe: how does this news article/ web page/ advert/ social media post make you feel? - Probe: what impact do you believe this news article/ web page/ advert/ social media post could have on someone’s behaviour? - Probe: who does this news article/ web page/ advert/ social media post stigmatise? - Probe: what is inaccurate about the news article/ web page/ advert/ social media post?   **Organisation trust**  X axis – how often do you read/ access information from this organisation/ publisher/ social media platform? Very often – never  Y axis – how much do you trust the health information provided by this organisation/ publisher/ social media platform?   - Probe: what makes them trustworthy/ untrustworthy? - Probe: have you always felt this way? - Probe: do you think other groups are more or less likely to trust them?   **Accessibility & quality of information**  X axis – how informative is the news article/ web page/ advert/ social media post? Very – Not at all  Y axis – how easy is it to understand the information? Very – Not at all   - Probe: is this information you already knew, or have you learnt something new? - Probe: what impact do you believe this news article/ web page/ advert/ social media post could have on someone’s behaviour? - Probe: what could be done to make the information more accessible to you/ others? - Probe: is there any key information missing? - Probe: how relevant is this information to you? | - Perceptions of existing Mpox messaging, its potential to be stigmatising, accessibility, relevance, accuracy - Level of trust in different organisations, news media, government, NHS, social media, NGOs/ community groups |
| Lunch | 12:00 – 13:00 |  | |
| Ask a question/ knowledge exchange | 13:00 – 13:30 | **10-20 minute presentation:**  Facilitator or clinician will give a presentation on key information relating to Mpox transmission, prevention and treatment. Information will reflect the latest evidence and practices, and sourced from trusted authorities.  **10-20 minute Q&A:**  What do you want to know about Mpox risk, treatment, prevention?  If answer to participant question is not known, the facilitator must state this and offer to conduct further investigation and respond to the participant at a later time. | Participant knowledge & information needs |
| Discussion: key challenges in Mpox prevention and treatment | 13:30 – 14:00 | Spilt participants into 2 groups.  Group 1 will discuss and write down key challenges in preventing Mpox.   - Probe: what increases people’s risk of contracting Mpox? - Probe: who is most at risk? - Probe: what challenges are there in accessing vaccines? - Probe: what challenges are there in accessing/ trusting public health information? - Probe: what challenges are there in self-isolation? - Probe: how do these challenges compare to those in HIV prevention & treatment?   Group 2 will discuss and write down key challenges in treating Mpox.   - Probe: what is preventing/ deterring people from seeking healthcare? - Probe: is diagnosis and treatment for Mpox accessible? - Probe: what challenges are there in accessing/ trusting public health information? - Probe: how do these challenges compare to those in HIV prevention & treatment?   After 15 minutes get the groups to switch and give them 15 minutes to discuss/ add to the other groups responses for prevention/ treatment. | Enablers and barriers to Mpox prevention & treatment, information needs |
| Communication strategy pitch | 14:00 – 15:00 | Working in the same groups as the previous activity, task the participants to develop a communication strategy to address one or more of the challenges identified during the previous activity. Instruct participants to use the following template to develop their strategy.  **Communication strategy goal** e.g., encourage people that have been exposed to Mpox to seek timely healthcare   - Probe: Are you trying to educate or provide new information? Are you calling your target audience to action? Are you trying to change behaviour? - Probe: will you goal be focused on Mpox, or will it relate to other infectious diseases?   **Target audience**   - Probe: what are their communication needs, preferences? - Probe: what is the best way to reach them? - Probe: are there any cultural, linguistic or literacy factors influencing the audience?   **Distribution mode(s)/ tactics:**   - Probe: will you use more than one communication platform? - Probe: what makes this distribution mode suitable for the target audience? - Probe: will you partner with any organisations?   **Key messages:**   - Probe: how will these messages help you to achieve the communication goal? - Probe: how can you frame the message in a way that is non-stigmatising/ accessible/ relevant to the target audience? - Probe: what distribution mode(s)/ tactic will be used to deliver the message?   Allow each group 30-40 minutes to develop and write down their strategy.  Give each group 5 minutes to pitch their strategy and 5 minutes Q&A. | Co-production of evidence based communication strategies |
| Closing statements | 15:00 – 15:30 | Closing statements from facilitator summarising some of the themes that emerged during the workshop.  Ask participants what they hope to see happen next.  Ask participants to complete a short workshop evaluation form. What they liked about the workshop and did not like? What recommendations they have to improve the workshop? What they take home from the workshop? | Key themes |
